# Complementary feeding practices and nutrient intakes of children aged 6–24 months from Bangladeshi background living in Tower Hamlets, East London: a feasibility study

**DOI:** 10.1186/s41043-020-0213-1

**Published:** 2020-02-28

**Authors:** Laura Jabri, Diana Margot Rosenthal, Lorna Benton, Monica Lakhanpaul

**Affiliations:** 1 Population, Policy and Practice Research and Teaching Department, UCL Great Ormond Street Institute of Child Health, 30 Guilford St, London, WC1N 1EH UK; 2 University College London, London, WC1E 6BT UK; 3 UCL Collaborative Centre for Inclusion Health, Department of Epidemiology and Public Health, London, WC1E 7HB UK

**Keywords:** Infant feeding, Complementary feeding practices, Bangladeshi, Nutrient intake

## Abstract

**Background:**

The aim of this study was to assess dietary intakes and complementary feeding practices of children aged 6–24 months who are from Bangladeshi ancestry and living in Tower Hamlets, London, and determine the feasibility of a larger, population-representative study.

**Methods:**

Questionnaires for demographic variables and feeding practices, and 24-h dietary recalls were administered to 25 mothers to determine whether it would be feasible to conduct a similar study on a representative sample size of the same population. Data from both tools were used to determine adequacy of complementary feeding practices through the WHO indicators and an infant and child feeding index score as well as overall macronutrient and micronutrient intake.

**Results:**

Four children had varying suboptimal complementary feeding practices: two children failed to achieve the minimum dietary diversity, one child was being fed cow’s milk before the age of 1 year, and one scored ‘poor’ on the infant and child feeding index. Most notably, the mean protein intake (39.7 g/day, SD 18.2) was higher than RNIs for all age groups (*P* = 0.001). Vitamin D intake was below recommendations (*P* = 0.006) for the 12–24-month age group. For the 10–12-month age group, zinc intake fell below recommendations (*P* = 0.028). For the 6–9-month combined age group, iron and zinc intakes were below recommendations (*P* = 0.021 and *P* = 0.002, respectively).

**Conclusions:**

Given the feasibility of this study, the results obtained require a large-scale study to be conducted to confirm findings. Our initial results indicated that children from Bangladeshi heritage may not be meeting nutritional requirements; thus, a future intervention tailored to the needs of the Bangladeshi population may be required to improve aspects of complementary feeding practices and nutrient intakes of those children.

## Background

Complementary feeding (CF) is the period of time when solid foods are introduced into the infant’s diet alongside breastfeeding because breast milk alone becomes insufficient to meet the growing nutritional needs of the infant [1]. Exclusive breastfeeding beyond 6 months of age causes growth faltering and malnutrition in infants [1, 2]. CF typically covers the range between 6 months and 2 years of age [3]. The first 1000 days of life, from conception until 2 years of age, are the most important stages of a person’s life in terms of growth and brain development [4]. Poor nutritional practices during this period, such as an early introduction of solid foods before 6 months, can lead to lifelong health consequences such as obesity and various non-communicable diseases including diabetes and cardiovascular disease [5]. In this manuscript, ‘weaning’ is synonymous with introducing complementary foods, which include the addition of any solid, semi-solid, or soft foods excluding breast milk, formula milk, juices, water, and other liquids [6].

Exclusive breastfeeding up to 6 months protects infants against infection [7, 8]. Introducing solid foods later than 6 months results in growth faltering and decreased rates of infant growth [9]. While both the World Health Organization (WHO) and National Health Service (NHS) currently recommend complementary feeding to start at 6 months, most infants in the United Kingdom (UK) are first given complementary foods before 5 months and only 22% have been introduced timely by 6 months [2, 10, 11]. Dietary diversity is also an important determinant of infant and young child health in both low-income and high-income countries [12, 13]. In Bangladesh, infants are given complementary foods that are primarily cereal-based, low in protein and micronutrients [14]. In the UK, the most common complementary foods are baby rice and pureed fruits or vegetables [10]. In general, research has linked the development of childhood obesity to inappropriate complementary feeding practices including low dietary diversity and very early introduction of complementary foods [9, 15, 16]. A large prospective cohort study using data from five low- and middle-income countries has also found that fast infant weight gain can contribute to adult excess weight, obesity, and hypertension [17].

Research assessing the current complementary feeding practices and their impact on infants and young children in Bangladesh is of unclear relevance to the context of high-income countries such as the UK. Little or no research has attempted to study the complementary feeding practices of South Asians in Europe or the UK. Recent national statistics from 2017 to 2018 show that obesity rates for Bangladeshi children in England climb up from 21% in 4–5 year olds to 44% in 10–11 year olds, placing the latter as the second highest obesity figure, preceded only by the Black-African ethnicity [18]. The complementary feeding practices of Bangladeshi parents in the UK are complex, since many factors influence the changes of an ethnic group’s dietary habits including acculturation, food availability, convenience, and income, all of which may contribute to the susceptibility for the development of various chronic diseases in adulthood [19]. Consequently, the feeding practices of British Bangladeshi children and adults alike in Tower Hamlets are regularly influenced by the UK society, the London Bengali community, and the Bangladeshi culture alike [20].

Most Bangladeshis in the UK reside in the Borough of Tower Hamlets in East London, where the median household income was £29,896 in 2016^1^ [21, 22]. The Bangladeshi community in Tower Hamlets has high rates of childhood obesity, diabetes, cardiovascular disease, and hypertension [23, 24]. The aims of this study were (1) to assess the current complementary practices and nutrient intakes of British Bangladeshi children ages 6–24 months living in Tower Hamlets and compare them with the recommendations by the WHO and NHS, which can provide insight into their possible contribution to childhood obesity and development of non-communicable diseases, and (2) to determine the feasibility of a larger, population-representative study and help towards highlighting the need to study other ethnic communities living in the UK and developing more tailored advice.

## Methods

### Study design and population

This cross-sectional study interviewed Bangladeshi mothers of children aged 6–24 months who were living in the East London Borough of Tower Hamlets between April and July 2016. The inclusion criteria included mothers from a Bangladeshi background who were (1) of reproductive age (18–49), (2) the primary caregivers of at least one child between the ages of 6–24 months, and (3) currently living in Tower Hamlets, London. Mothers suffering from mental illnesses or substance abuse and children suffering from any long-term chronic conditions or disabilities were not included in the study. Due to limited resources to hire a certified interpreter, only English-speaking mothers were recruited. Participants were recruited by convenience and snowball sampling through a Bangladeshi community facilitator, who was part of the Nurture Early for Optimal Nutrition (NEON) study, a participatory female health volunteer led intervention to promote healthy nutrition in children of Bangladeshi origin in East London [25]. Participants attended individual face-to-face interview sessions during which descriptive quantitative data about current complementary feeding practices were obtained through a paper-based demographic questionnaire, and dietary intakes were obtained through a quantitative 24-h dietary recall (24hDR). To account for illiteracy or lower literacy levels of some participants, the information was obtained from the participants verbally and recorded by the researcher during each interview. Each participant received £20 incentive vouchers as time-and-travel compensation.

This study was approved by UCL Research Ethics Committee (8551/001), and written informed consent was obtained from all participants. All data were collected, stored, and handled in accordance with the provisions of the Data Protection Act 1998 and 2018. All data were anonymised and used for this academic study only.

### Sociodemographic and anthropometric variables

Sociodemographic information including maternal age, years of residence in the UK, maternal and paternal education levels, and total household income was collected using the questionnaire, which also included a series of questions about feeding practices. The mothers’ weights and heights were measured during the interview session in order to test a potential association between maternal BMI and child birthweight [26]. Height was measured to the nearest 0.5 cm using the Leicester Height Measure HM250P (Marsden, UK). Weight was measured to the nearest 100 g using the Body Composition Monitor BF500 (Omron, The Netherlands). Participants were weighed barefoot and with light clothing. Birthweight in addition to the most recent weight and height of subject infants and children was obtained from the Personal Child Health Record (PCHR). An estimation of the child’s weight at the time of interview was calculated using the WHO’s weight velocity standards [27].

### Assessment of dietary intake

The 24hDR is a common tool used in many studies to assess the dietary intake of infants and young children [28]. This tool is useful because of its short administration time and low need for respondent literacy. However, it is still subject to respondent and recall bias, and may not capture usual intake [29]. Other advantages and drawbacks of the 24hDR have been discussed elsewhere [29–32]. This tool was chosen because it has been validated for use in European populations of different ethnicities to assess average intake and can adequately assess intake among infants and children 4–24 months of age [29, 33]. The WHO uses the 24hDR to derive core and optional indicators of complementary feeding practices that have been widely used in developing countries [6, 34, 35]. Probing questions for accuracy in estimation of dietary intake for the 24hDR were used during the interview [35]. Mothers were asked to estimate portion sizes by referring to a displayed chart of common household measures (e.g. cup, tablespoon) for food estimation and a standard 200-ml cup for beverage estimation.

### Assessment of complementary feeding practices

Feeding practices were assessed using data from the 24hDR and questions from the demographic questionnaire such as age of introduction of CF and early initiation of breastfeeding. The WHO indicators used in this study were early initiation of breastfeeding, bottle-feeding, introduction of complementary foods, consumption of iron-rich or iron-fortified foods, minimum dietary diversity (MDD), minimum meal frequency (MMF), and minimum acceptable diet (MAD). MDD was assessed with regard to intake of 7 food groups: (1) grains, roots, and tubers; (2) legumes and nuts; (3) dairy products; (4) flesh foods; (5) eggs; (6) vitamin A-rich fruits and vegetables; and (7) other fruits and vegetables. All milk sources including formula but excluding breast milk were counted in the dairy products food group [6]. Calculating the MMF values was based on the Feeding Infants and Toddlers Study methodology [36]. Every meal or snack was counted except if consumed in trivial amounts (< 5 g). The feeding frequency of the breastfed children excluded the breast milk feeds. MMF for breastfed infants was 2 times and 3 times for 6–8 months and 9–23 months, respectively, and for the non-breastfed infant 6–23 months, 4 times [36]. Another useful tool to assess the overall quality of complementary feeding practices used in this study was the infant and child feeding index (ICFI) [37]. A modified version of the ICFI was used in this study because it has the ability to provide information about the quality of complementary foods without the need for a 7-day food frequency questionnaire. The index attributes scores for different components including breastfeeding, food variety, and meal and snack frequency for a total range of 0–9 points. The index score was validated and found to be positively correlated with better anthropometric parameters. Details of the components and scoring system of the modified ICFI have been stated elsewhere [38].

### Statistical analysis

The 24hDR data was analysed using the DietPlan6 (2003) software for Windows (Forestfield Software Ltd., UK) for macronutrient and micronutrient intake. Converting food portion sizes to estimated weight measures in grammes to be entered into Dietplan6 was carried out using several sources [39–43]. Dietary supplements were excluded from nutrient intake analysis. Estimation of breastfeeding quantities was adapted from Feeding Infants and Toddlers Study methodology [44]. Each breastfeeding instance in the 24hDR was considered as a feeding and calculated based on the child’s age, breastfeeding status, and amount of other milk sources reported in the recall.

Macronutrients of interest in this study were total caloric intake, protein, total carbohydrates, total sugars, total fats, and saturated fats. Several micronutrients were chosen due to their higher prevalence of deficiencies: iron, zinc, calcium, vitamin A, vitamin C, vitamin D, thiamine, riboflavin, niacin, vitamin B6, and folate [35]. Sodium intake was also included because it has been highly correlated with hypertension [45]. Vitamin A nutrient was calculated according to the following equation [46]:

$$ Vitamin\ A\ \left(\mu g\right)= retinol\ \left(\mu g\right)+\left(\beta - carotene\ \left(\mu g\right)/6\right) $$

One-sample *t* tests, paired samples *t* test, independent samples *t* test, one-way ANOVA, and Pearson’s correlations were used for various analyses of the data. Although some variables were not normally distributed (child’s age, fat, calcium, vitamin A, vitamin D, thiamin, riboflavin, niacin, folate), outliers were not removed because of small sample size and were deemed reasonable upon inspection. Although some variables were not normally distributed, the parametric *t* tests were still valid for non-normally distributed data [47], so they were still chosen as the test of choice. *P* values < 0.05 were considered statistically significant. Statistical analyses were carried out in Microsoft Excel 2016 for Mac (Microsoft, USA) and the IBM SPSS Statistics Software version 22 (IBM Analytics, USA).

## Results

### Sociodemographic characteristics

**Fig. 1:**
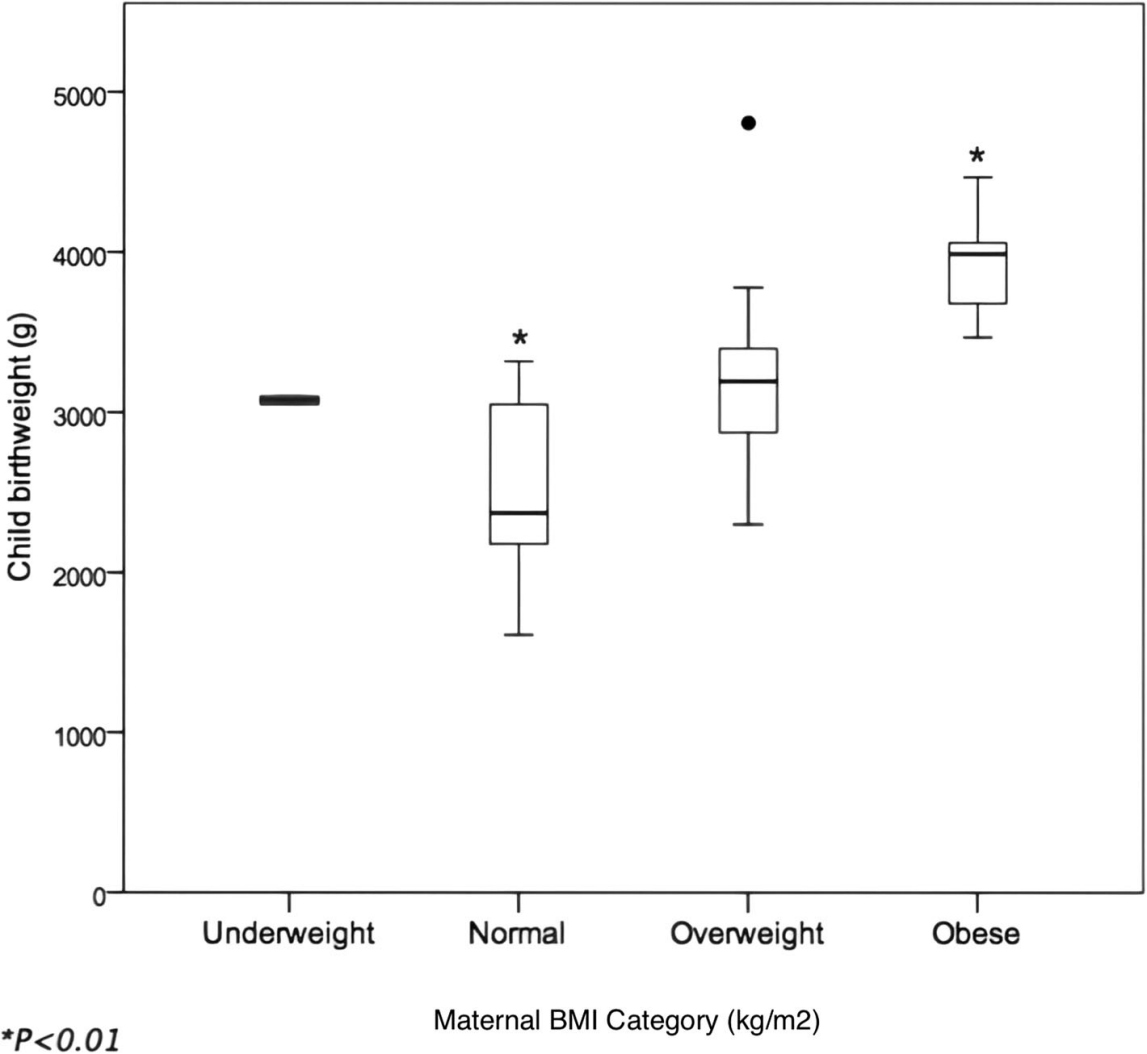
Maternal BMI category and child birthweight

Twenty-five mothers with a mean age of 31.5 years (standard deviation (SD) = 4.1) were interviewed. Subject children had a mean age of 13.5 months (SD = 5.8). All mothers were born in Bangladesh, but only four (16%) mothers had lived in the UK for 3 years or less, with a range of 2–31 years. All mothers were married at the time of the interview and practiced Islam as a religion. Educational level of both parents was high overall, with only three fathers and three mothers having an education below high school. The mean age of mothers at their first childbirth was 25.3 years (SD = 3.8). Although this sample was not population-representative due to time and financial constraints, sociodemographic and dietary characteristics compare well with other representative figures and data. Sociodemographic, anthropometric, and pregnancy and childbirth characteristics of the sample were collected and analysed (Tables 1, 2, and 3). Maternal anthropometric characteristics are reported in detail in an additional table (see Additional file 1). Birthweights of children were significantly different between the normal (*n* = 6, mean = 2483.3, SD = 628.3) and obese (*n* = 5, mean = 3934.0, SD = 382.8) maternal body mass index (BMI) scores (*P* = 0.004) (Fig. 1).

**Table 1:** Selected sociodemographic characteristics of sample population (*n* = 25)

Characteristic	Number	Percentage
Household		
Housing type		
Owned	3	12
Rented	15	60
Council house	7	28
Monthly household income		
< £1000	7	31.8
£1000–£1499	11	50
£1500–£2499	3	13.6
£2500–£3499	1	4.5
Extended family living in household		
Yes	7	28
No	18	72
Mother		
Maternal education		
Primary	1	4
Secondary	2	8
High school	6	24
College	7	28
Postgraduate	9	36
Residence in the UK		
3 years or less	4	16
> 3 years	21	84
Working mother		
Yes	17	68
No	5	20
Student	3	12
Father		
Paternal education		
Primary	–	–
Secondary	3	12
High school	–	–
College	11	44
Postgraduate	11	44

**Table 2:** Subject child characteristics (*n* = 25)

Characteristic	Number	Percentage
Age group		
6–8 months	6	24
9–11 months	6	24
12–24 months	13	52
Gender		
Male	13	52
Female	12	48
Birth order		
1st born	10	40
2nd born or higher	15	60
Child attending day care		
Yes	2	8
No	23	92
Average child illness per month		
< 1 time	14	56
1–2 times	10	40
3–4 times	1	4

**Table 3:** Pregnancy and childbirth characteristics (*n* = 25)

Characteristic	Mean	SD
Birthweight (g)	3186.4	724.4
Gestational age (weeks)	35.8	10.9
	*N*	%
Type of delivery		
Vaginal	17	74
Caesarean	6	26
Not indicated	2	8
Low birthweight*		
Yes	4	16
No	21	84
Pregnancy complications		
None	11	44
Gestational diabetes	6	24
Preterm birth	2	8
Small for gestational age^†^	2	8
Other^‡^	8	32

*SD* standard deviation

*Definition criterion uses < 2500 g [48]

^†^Definition criterion uses birthweight < 10th centile [48]

^‡^Individual cases include intra-uterine growth restriction (*n* = 1), hypothyroidism (*n* = 2), hypertension (*n* = 1), neonatal hypoglycaemia (*n* = 1), rubella non-immune (*n* = 1), no placental support post 7-month gestation (*n* = 1), and breech spontaneous vaginal delivery (*n* = 1)

### Complementary feeding practices

More than half the children (56%) were mixed-feeding (Table 4). Children classified as mixed-fed include infants who received at least one feeding or one instance of either breast milk or formula milk. Five infants were classified under mixed-fed given formula milk in hospital but continued to be exclusively breastfed at home. Out of 14 mothers who were breastfeeding, 7 were also giving bottle-feeds to their children. No mother introduced complementary feeding before 4 months or after 8 months of her child’s age. The supplements given to the children were Healthy Start (*n* = 18), Well Baby (*n* = 2), and Abidec (*n* = 1).

**Table 4:** Feeding practices of sample population (*n* = 25)

Feeding practice	Number	Percentage
Feeding method 0–6 months of age		
Exclusive breastfeeding*	2	8
Predominant breastfeeding*	1	4
Complementary feeding*	7	28
Mixed feeding^†^	14	56
Formula feeding^‡^	1	4
Early initiation of breastfeeding^§^		
Yes	10	45
No	12	55
Bottle feeding^||^		
Yes	18	72
No	7	28
Introduction of complementary foods		
< 6 months	7	28
6 months	15	60
> 6 months	3	12
Age of introduction of formula milk		
< 1 month	15	60
1–3 months	4	16
> 3 months	6	24
Whole cow’s milk given as drink < 1 year of age^¶^		
Yes	1	4
No	24	96
Currently breastfeeding		
Yes	14	56
No	11	44
Supplement use		
Yes	21	84
No	4	16

*According to WHO definition [6]

^†^Allows infant to receive breast milk and non-human milk or formula milk, but not complementary foods

^‡^Infant exclusively formula feeding; has never been breastfed

^§^Early initiation of breastfeeding: < 60 min after birth

^**||**^Child who received any food or drink from a bottle during the previous day

^**¶**^NHS recommendation: whole cow’s milk can be mixed with foods or given as yogurt or fromage frais after 6 months of age; whole cow’s milk can be given as a drink from 1 year of age [11]

Two mothers answered ‘Yes’ to currently breastfeeding, but no breastfeeds were reported in the 24hDR of the previous day (Table 5). Formula-feeding rates increased from 6–8 months to 9–11 months but dropped during 12–24 months due to replacement with whole cow’s milk. Bottle-feeding rates also increased from 12 months onwards. All children who were reported to be breastfeeding, but were not breastfed the previous day, were considered non-breastfed based on the WHO indicators (Table 6). Indicators were not separated according to different age groups due to the small sample size. One breastfed and one non-breastfed child did not meet the minimum dietary diversity. One child had not consumed any iron-rich or iron-fortified foods the previous day.

**Table 5:** Selected feeding practices stratified by age (*n* = 25)

	Age group		
	6–8 months (*n* = 6)	9–11 months (*n* = 6)	12–24 months (*n* = 13)
Currently breastfeeding (%)	83.0	50.0	46.2
Formula feeding (%)	50.0	66.7	23.1
Consumption of cow’s milk (%)	0.0	16.7	84.6
Bottle feeding (%)	66.7	66.7	81.8

**Table 6:** WHO indicators of complementary feeding practices

Indicator	Percentage	
Introduction of solid, semi-solid, or soft foods (*n* = 6)	100	
Consumption of iron-rich or iron-fortified foods (*n* = 25)	96	
	Breastfed (*n* = 12)	Non-breastfed (*n* = 13)
Minimum dietary diversity	92	92
Minimum meal frequency	100	100
Minimum acceptable diet	92	92

As per WHO definition [6]

**Fig. 2:**
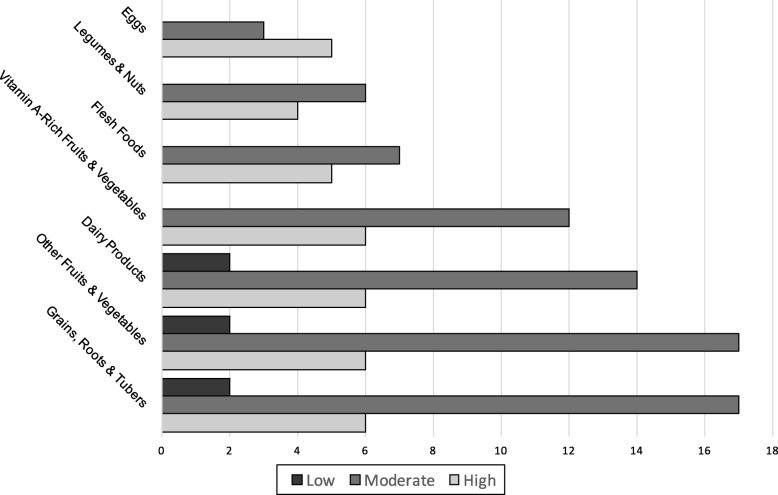
Categorised dietary diversity scores and intake frequency of each food group

Foods given to the children during the previous day included some Bangladeshi ethnic foods such as kichuri or dhaal, baby cereals, rice porridge, different fruits and vegetables, commercial fruit yogurts, and various snacks. Home-made foods usually consisted of rice mixed with several different vegetables and sometimes beans or lentils. Recipes for kichuri included red lentils for some participants but not for others. All children had eaten from the grains and fruits and vegetable groups, 88% had dairy products, 72% had vitamin A-rich fruits and vegetables, 48% had flesh foods, 40% had legumes and nuts, and 32% had eggs. The dietary diversity scores were classified as low, moderate, and high when 0–3, 4–5, and 6–7 food groups were consumed throughout the day, respectively (Fig. 2).

**Fig. 3:**
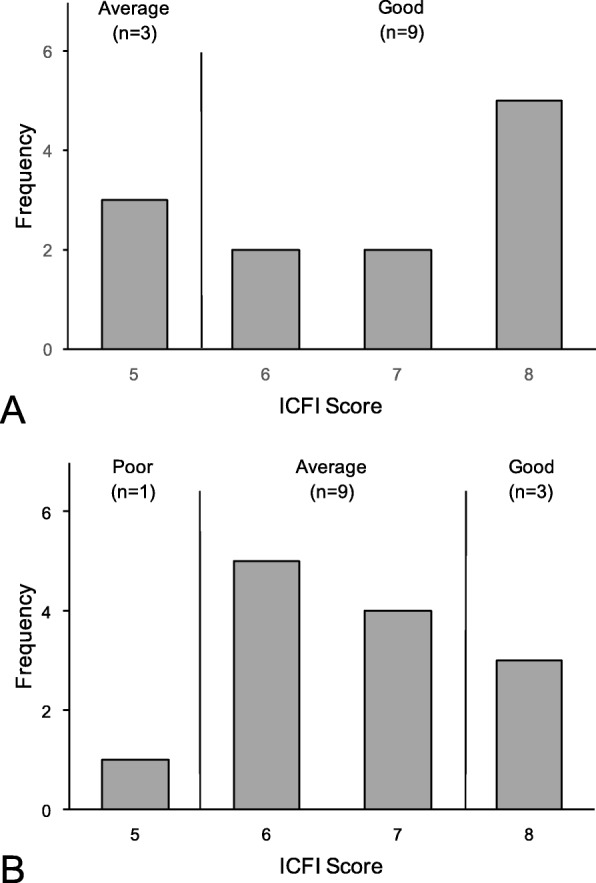
ICFI score category and frequency for children aged **a** 6–11 months and **b** 12–24 months. ICFI, infant and child feeding index

One child out of 25 in the 12–24 age group had a low score on ICFI (Fig. 3). This child had eaten rice pudding, dhaal, pita bread, and fruit yogurt in addition to cow’s milk the previous day. There were no significant differences among the ICFI categories with the child’s age (*P* = 0.991), maternal age (*P* = 0.644), estimated current body weight (mean = 9.47 kg, SD = 1.82) (*P* = 0.515), estimated energy intake (*P* = 0.818), estimated protein intake (*P* = 0.556), energy intake from protein (*P* = 0.291), iron intake (*P* = 0.427), zinc intake (*P* = 0.727), calcium intake (*P* = 0.854), vitamin C intake (*P* = 0.462), or vitamin D intake (*P* = 0.170) using one-way analysis of variance (ANOVA). Moreover, there were no significant correlations between the ICFI categories and household income (*P* = 0.936) or maternal educational level (*P* = 0.410) with Pearson’s test.

### Dietary intake

Nutrient intakes of the children were stratified by age group (Tables 7, 8, 9, 10, and 11). Because only two children were in the 6-month age group (Table 7), they were combined with the 7–9-month age group in a separate table (Table 9) for larger sample size analysis. Overall, several micronutrient intakes were below the UK Dietary References Values (DRVs) for various age groups including zinc, iron, vitamin D, riboflavin, and niacin. Other nutrients including calcium, thiamine, folate, and vitamin C intakes were higher in several age groups. The mean protein intake of the oldest age group was 274% higher than the recommendation, even though 8.3% were not meeting DRV. The mean energy intake was not significantly different from the estimated average requirements for the different feeding groups (breastfed, formula-fed, and mixed-fed) categorised by gender and age (Table 12). Total sugar intake of the sample population has a mean of 27.7% (SD = 1.8) of total energy intake (Table 13).

**Table 7:** Nutrient intakes from diet of the 4–6-month age group (*n* = 2) compared with dietary reference values (DRVs)

Nutrient	DRV	Mean	SD	Percentage of DRV	Percentage of sample not meeting DRV	Minimum	Maximum	*P* value*
Iron (mg/day)	4.30	2.5	2.4	58.8	100.0	0.8	4.2	0.489
Zinc (mg/day)	4.0	2.8	0.8	69.3	100.0	2.2	3.3	0.276
Calcium (mg/day)	525.0	262.0	43.8	49.9	100.0	231.0	293.0	0.075
Sodium (mg/day)	280.0	242.0	206.5	86.4	50.0	96.0	388.0	0.838
Vitamin A (μg/day)	350.0	1027.2	924.0	293.4	0.0	373.8	1860.5	0.489
Vitamin D (μg/day)	8.5	0.6	–	6.5	100.0	0.6	0.6	–
Thiamin (mg/day)	0.2	0.3	0.2	165.0	0.0	0.2	0.5	0.500
Riboflavin (mg/day)	0.4	0.3	0.0	65.0	100.0	0.3	0.3	0.022
Niacin (mg/day)	3.0	3.2	1.7	107.0	50.0	2.0	4.4	0.893
Vitamin B6 (mg/day)^†^	0.2	0.3	1.0	145.0	0.0	0.2	0.4	0.421
Folate (μg/day)	50.0	116.0	103.2	232.0	50.0	43.0	189.0	0.532
Vitamin C (mg/day)	25.0	49.0	21.2	196.0	0.0	34.0	64.0	0.356
Protein (g/day)	12.7	15.5	8.8	122.1	50.0	9.3	21.7	0.730

Nutrient intakes from diet excluded dietary supplements

*P value for differences between mean intake of children in the sample from the specified age group and the respective DRV

^†^Based on protein providing 14.7% of EAR for energy

**Table 8:** Nutrient intakes from diet of the 7–9-month age group (*n* = 5) compared with dietary reference values (DRVs)

Nutrient	DRV	Mean	SD	Percentage of DRV	Percentage of sample not meeting DRV	Minimum	Maximum	*P* value*
Iron (mg/day)	7.8	5.8	2.1	74.4	80.0	2.9	8.7	0.097
Zinc (mg/day)	5.0	3.8	0.6	76.0	100.0	2.9	4.4	0.010*
Calcium (mg/day)	525.0	449.2	168.6	85.6	60.0	286.0	661.0	0.372
Sodium (mg/day)	320.0	385.2	393.3	120.4	80.0	122.0	1081.0	0.730
Vitamin A (μg/day)	350.0	377.7	248.1	107.9	40.0	48.0	718.3	0.815
Vitamin D (μg/day)	7.0	4.2	3.4	59.9	60.0	0.3	7.6	0.136
Thiamin (mg/day)	0.2	0.5	0.2	235.0	0.0	0.3	0.7	0.032*
Riboflavin (mg/day)	0.4	0.8	0.3	195.0	0.0	0.6	1.4	0.061
Niacin (mg/day)	4.0	5.1	2.9	126.3	60.0	2.7	9.4	0.460
Vitamin B6 (mg/day)^†^	0.3	0.45	0.3	160.0	40.0	0.2	1.0	0.286
Folate (μg/day)	50.0	109.2	36.3	218.4	0.0	65.0	160.0	0.022*
Vitamin C (mg/day)	25.0	66.4	5.9	265.6	0.0	60.0	74.0	0.000*
Protein (g/day)	13.7	16.3	4.8	118.9	20.0	8.3	20.5	0.286

Nutrient intakes from diet excluded dietary supplements

*P value for differences between mean intake of children in the sample from the specified age group and the respective DRV

^†^Based on protein providing 14.7% of EAR for energy

**Table 9:** Nutrient intakes from diet of the combined 6–9-month age (*n* = 7) compared with dietary reference values (DRVs)

Nutrient	DRV	Mean	SD	Percentage of DRV	Percentage of sample not meeting DRV	Minimum	Maximum	*P* value*
Iron (mg/day)	7.8	4.8	2.5	61.9	75.0	0.8	8.7	0.021
Zinc (mg/day)	5.0	3.5	0.8	70.0	87.5	2.2	4.4	0.002
Calcium (mg/day)	525.0	395.7	166.2	75.4	62.5	231.0	661.0	0.085
Sodium (mg/day)	320.0	344.3	339.3	107.6	62.5	96.0	1081.0	0.856
Vitamin A (μg/day)	350.0	563.3	532.7	160.9	25.0	48.0	1680.5	0.330
Vitamin D (μg/day)	7.0	3.6	3.4	51.3	50.0	0.3	7.6	0.055
Thiamin (mg/day)	0.2	0.4	0.2	215.0	0.0	0.2	0.7	0.016
Riboflavin (mg/day)	0.4	0.6	0.4	157.5	25.0	0.3	1.4	0.151
Niacin (mg/day)	4.0	4.5	2.6	112.5	50.0	2.0	9.4	0.615
Vitamin B6 (mg/day)^†^	0.3	0.4	0.3	140.0	37.5	0.2	1.02	0.289
Folate (μg/day)	50.0	111.1	51.7	222.2	12.5	43.0	189.0	0.020
Vitamin C (mg/day)	25.0	61.4	13.1	245.6	0.0	34.0	74.0	0.000
Protein (g/day)	13.7	16.1	5.3	117.5	25.0	8.3	21.7	0.278

Nutrient intakes from diet excluded dietary supplements

*P value for differences between mean intake of children in the sample from the specified age group and the respective DRV

^†^Based on protein providing 14.7% of EAR for energy

**Table 10:** Nutrient intakes from diet of the 10–12-month age group (*n* = 6) compared with dietary reference values (DRVs)

Nutrient	DRV	Mean	SD	Percentage of DRV	Percentage of sample not meeting DRV	Minimum	Maximum	*P* value*
Iron (mg/day)	7.8	5.4	4.1	69.2	83.3	1.2	12.5	0.203
Zinc (mg/day)	5.0	3.2	1.5	64.0	83.3	2.0	5.9	0.028
Calcium (mg/day)	525.0	434.7	290.7	82.8	66.7	207.0	959.0	0.481
Sodium (mg/day)	350.0	391.8	319.6	111.9	66.7	131.0	1020.0	0.761
Vitamin A (μg/day)	350.0	331.4	263.0	94.7	33.3	2.3	659.5	0.869
Vitamin D (μg/day)	7.0	4.4	4.3	62.9	66.7	0.9	9.2	0.398
Thiamin (mg/day)	0.3	0.5	0.34	173.3	33.3	0.2	1.2	0.214
Riboflavin (mg/day)	0.4	0.7	0.5	175.0	50.0	0.3	1.4	0.203
Niacin (mg/day)	5.0	4.8	3.2	96.0	66.7	1.7	9.5	0.020
Vitamin B6 (mg/day)^†^	0.4	0.5	0.4	122.5	66.7	0.1	1.1	0.585
Folate (μg/day)	50.0	102.5	79.7	205.0	16.7	38.0	240.0	0.168
Vitamin C (mg/day)	25.0	46.8	30.9	187.2	33.3	21.0	104.0	0.144
Protein (g/day)	14.9	18.5	8.2	124.2	50.0	10.5	31.6	0.335

Nutrient intakes from diet excluded dietary supplements

*P value for differences between mean intake of children in the sample from the specified age group and the respective DRV

^†^Based on protein providing 14.7% of EAR for energy

**Table 11:** Nutrient intakes from diet of the 12–24-month age group (*n* = 12) compared with dietary reference values (DRVs)

Nutrient	DRV	Mean	SD	Percentage of DRV	Percentage of sample not meeting DRV	Minimum	Maximum	*P* value*
Iron (mg/day)	6.9	7.5	5.0	108.6	66.7	2.2	21.3	0.681
Zinc (mg/day)	5.0	4.3	1.4	86.0	66.7	2.0	7.3	0.136
Calcium (mg/day)	350.0	609.9	315.8	174.3	16.7	139.0	1307.0	0.016
Sodium (mg/day)	500.0	808.7	558.0	161.7	33.3	284.0	2043.0	0.082
Vitamin A (μg/day)	400.0	680.6	815.1	170.2	58.3	0.0	2690.2	0.258
Vitamin D (μg/day)	7.0	2.5	3.7	35.7	77.8	0.0	9.6	0.006
Thiamin (mg/day)	0.5	0.8	0.6	154.0	33.3	0.3	2.4	0.132
Riboflavin (mg/day)	0.6	1.3	0.7	220.0	16.7	0.3	2.8	0.004
Niacin (mg/day)	8.0	8.5	8.1	106.3	75.0	2.5	32.4	0.829
Vitamin B6 (mg/day)^†^	0.7	0.7	0.3	105.7	41.7	0.3	1.1	0.683
Folate (μg/day)	70.0	157.6	84.3	225.1	0.0	74.0	352.0	0.004
Vitamin C (mg/day)	30.0	66.8	51.4	222.7	33.3	6.0	172.0	0.030
Protein (g/day)	14.5	39.7	18.2	273.8	8.3	11.0	84.3	0.001

Nutrient intakes from diet excluded dietary supplements

*P value for differences between mean intake of children in the sample from the specified age group and the respective DRV

^†^Based on protein providing 14.7% of EAR for energy

**Table 12:** Energy estimated average requirements (EAR) for infants and children stratified by age, gender, and breastfeeding status

		Breastfed				Formula-fed				Mixed-fed or unknown			
		EAR	Intake		*P*	EAR	Intake		*P*	EAR	Intake		*P*
			Mean	SD			Mean	SD			Mean	SD	
7–12 months	Males	694.0	754.7	186.0	0.629	742.0	733.3	343.0	0.969	718.0	547.5	139.9	0.093
	Females	646.0	636.0^†^	–	–	670.0	–	–	–	646.0	580.5	101.1	0.528
1 year‡	Males	–	–	–	–	–	–	–	–	765.0	820.7	201.1	0.679
	Females	–	–	–	–	–	–	–	–	717.0	845.8	237.7	0.143

*SD* standard deviation

Based on the UK nutrition requirements [49]

^†^One value in category. Standard deviation and *P* value could not be obtained

^‡^EAR for male and female infants do not contain breastfed and formula-fed energy requirements for ages beyond 12 months. Mixed-fed was used in place

**Table 13:** Caloric intake, macronutrient intake, and percentages of total energy intake

	Daily intake		Min.	Max.	95% CI	Percentage of caloric intake		Min.	Max.	95% CI
	Mean	SD				Mean	SD			
Calories (kCal/day)	730.3	68.9	392.0	1210.0	578.6, 882.0	–	–	–	–	–
Calories from BF (kCal/day)	269.4	42.7	59.0	414.0	175.5, 363.4					
Calories from CF (kCal/day)	460.9	85.0	127.0	1093.0	273.8, 648.1					
Protein (g/day)	27.8	6.4	8.3	84.3	13.7, 41.8	13.7	1.9	6.4	27.9	9.6, 17.4
Total carbohydrates (g/day)	88.6	9.9	53.1	151.7	66.8, 110.4	48.2	1.8	40.7	61.7	44.2, 52.2
Total sugars (g/day)	52.9	3.6	33.7	69.3	44.9, 60.9	27.7	1.8	14.1	49.1	23.9, 31.4
Total fat (g/day)	29.9	1.7	17.8	39.3	26.1, 33.6	38.5	2.2	24.1	49.2	33.8, 43.3
Saturated fats (g/day)	13.4	1.1	6.1	22.5	10.9, 15.8	15.5	0.9	7.2	25.4	13.5, 17.5

*SD* standard deviation

No DRVs have been set for carbohydrate and fat intakes for infants and children younger than 2 years of age [46]

## Discussion

To our knowledge, the complementary feeding practices and dietary intake of infants and children aged 6–24 months had not been previously explored within the Bangladeshi population of Tower Hamlets. This study reported on complementary feeding practices and nutrient intakes of 25 Bangladeshi children aged 6–24 months living in Tower Hamlets to determine the feasibility of a larger study. The Tower Hamlets Bangladeshi population has high rates of child and adult obesity and chronic disease; thus, an investigation of early feeding practices was warranted. Although our sample size was limited, it was reflective of the Tower Hamlets population as evidenced by the similar income range between both. Moreover, the mothers interviewed were all non-UK born, which highly corresponds to the 2011 Census analysis [50]. The complementary feeding practices and dietary intakes were assessed using WHO indicators, an ICFI, and a 24hDR. This combination provided valuable information about the quality of complementary feeding practices and dietary intakes. We found that complementary feeding practices of 4 children out of 25 fall short from the UK DRVs. For different age groups, iron, zinc, and vitamin D intakes were lower than the RNIs, while calcium, vitamin C, folate, thiamine, and riboflavin intakes were higher. The mean sugar intake as a percentage of total energy intake for all children was 27.7%. Obese maternal BMI was associated with higher child birthweight compared to normal maternal BMI.

### Implications

In a 2011 study, the UK Department of Health and Food Standards Agency reported only 22% of children were introduced to complementary foods at 6 months, while 75% were introduced before 5 months [10]. Our findings indicated better adherence to early weaning than to late weaning. Additionally, 16% of our sample gave cow’s milk between 8 and 11 months of the child’s age. In Bangladesh, cow’s milk was commonly given as an early complementary food and perceived by some mothers as a good complementary food before 1 year of age [51]. On the other hand, national data from the UK shows that only 4% of mothers gave their child cow’s milk by 8–10 months of age [52], which corresponds better to our findings. Cow’s milk contains higher levels of protein and minerals and lower vitamin C than breast milk; guidelines from Australia and the ESPGHAN committee advise against its consumption before 12 months of age, and a systematic review found that this behaviour was associated with low iron status in infants and toddlers [53–55]. While these guidelines are commonly relayed by the NHS to mothers in the UK, the same cannot be said about the health services in Bangladesh, which might help explain the differing practice of cow’s milk feeding between both countries. Regarding breastfeeding, the discrepancy between mothers who confirmed breastfeeding but did not report instances of breastfeeding the previous day can be explained by their understanding of the question—it is possible that mothers still breastfed their child but not daily.

The ICFI, higher dietary diversity, and food variety scores were all found to be positively associated with height-for-age and weight-for-age *z*-scores in previous studies, consequently suggesting that poorer diet quality can hinder child optimal growth and development [38, 56]. However, the ICFI and WHO indicators have not been used in high-income countries such as the UK, but rather used in low-income settings and countries like Bangladesh. While we were not able to compare with height-for-age or weight-for-height *z*-scores as they were not available, we found no particular association of the ICFI scores and estimated current body weight. Two children in our study failed to achieve minimum dietary diversity (≥ 4) suggesting a lower diet quality and thus limited nutrient intakes [57]. Long-term diet quality largely affects nutritional status and anthropometric measurements of children, and although the WHO indicators and ICFI capture a single 24-h dietary intake, they can be informative regarding poorer complementary feeding practices that could be improved [38]. This is especially important given the high child obesity rates of the Bangladeshi population in England compared to other ethnic groups [18].

Dietary intakes of our sample were very similar to intakes of children from the Gemini Twin Cohort study in the UK [58]. All children in our sample had higher protein intakes than recommended. Some research suggests that high protein intakes during infancy and childhood increase the risk of developing obesity and non-communicable diseases in adulthood although the strength of evidence remains unclear [55, 59]. Positive associations were found between high protein intake and body fat percentage, waist circumference, higher BMI, and weight scores in later childhood [60, 61]. Protein intake could potentially be causing a general increased risk of childhood obesity in the UK [58].

Similar to results observed in our study, a trend could be established regarding the suboptimal intakes of several nutrients including zinc, vitamin A, and riboflavin among South Asian children of different age groups [62, 63]. Compared to white Europeans, South Asian children in the UK had higher intake of total energy, fat, protein, and starch but lower intake of sugar and micronutrients [64]. Results from the nationally representative diet and nutrition survey of infants and young children (DNSIYC) in the UK showed that 28% of the 12–18-month-old South Asian children had low iron intakes [10], similar to observations in our sample. For a 9–11-month-old infant, complementary foods must provide most zinc and iron requirements [65]. Meat is an excellent source of iron and zinc [66], but its consumption was low in our sample, which may explain the low zinc and iron status of some age groups. Low vitamin D intake was also found for some age groups in our sample, which has been linked to increased risks of cardiovascular disease and type 1 diabetes [67]. Vitamin D deficiency has been linked to impaired glucose tolerance in adult Bangladeshis of East London [68]. Higher intakes of calcium and several vitamins may warrant more attention regarding possible risks they may impose on the Bangladeshi population. The UK Department of Health recommends giving all infants vitamin A, C, and D supplements beyond 6 months of age, except those consuming 500 ml of formula each day [11]. All children who were formula-fed more than 500 ml daily in this sample were given supplements nonetheless. Based on these results, it is possible that the children benefited only from the vitamin D, and government recommendation should be reviewed. On another note, several maternal parameters could be indirectly contributing to child overweight and obesity and risk of chronic disease. Firstly, pre-pregnancy maternal BMI could be contributing to the increased birthweights of infants, which may be persist into childhood and adulthood as overweight [69–71]. Moreover, there is an association between a Caesarean delivery and higher incidence of overweight and chronic disease for the children [72, 73]. It may be possible to counsel mothers about improving their weights prior to pregnancy, and if possible, choose a vaginal delivery, in order to ensure healthier outcomes for their children. Last but not least, there are many economic and social factors that can contribute to health inequalities among low-income families and minority groups from various ethnic backgrounds in the UK. Such inequalities that are prevalent in the Bangladeshi population in Tower Hamlets place that community at increased risks of child obesity, adult obesity, and a range of chronic diseases compared to less-deprived areas [74]. The increased prevalence of childhood obesity and various adult chronic diseases in the Tower Hamlets Bangladeshi population requires investigation of the diet quality of infants and children 6–24 months to determine whether interventions are needed for this age group, since early intervention is essential for the prevention of obesity and long-term chronic health complications.

### Study feasibility

This study established the feasibility of large-scale research assessing current complementary feeding practices and nutrient intakes of Bangladeshi children in Tower Hamlets. The WHO indicators provide important information regarding dietary quality that may not be directly apparent from assessing nutrient intakes especially for our target population. Conversely, the use of those tools alone, while good proxies for dietary quality, may not provide sufficient understanding of the actual nutrient intakes of children. Several suboptimal feeding practices were identified, which were rather important findings for a high-income setting such as the UK, since the tools used were primarily developed for use in low-income countries. Nutrient intakes of infants and young children 6–24 months of age were especially important given the critical growth phase they are experiencing, and an imbalance could be causing various adverse health effects that persist into adulthood. In particular, the combination of low vitamin D and high sugar with the protein intakes of the children in our sample suggested a possible risk factor for diabetes and obesity in the Bangladeshi population. Finally, although no demographic factors were found to be predictors of nutrient intakes or complementary feeding practices, such associations may become apparent in a larger study.

A complementary feeding utility index (CFUI) [75] has been developed to assess complementary feeding practices of high-income countries; however, it requires longitudinal follow-up and its applicability to lower-income ethnic groups is questionable. Some components of the CFUI measure intakes of the increasingly popular energy-dense, nutrient-poor foods which are not emphasised in the ICFI. A future recommendation would be to validate a more simplistic tool such as the ICFI for cross-sectional studies in high-income countries that would also reflect the sociodemographic characteristics and changing dietary habits of ethnic groups with focus on components that are more relevant to health outcomes such as obesity and non-communicable diseases.

### Limitations

This is the first study that we are aware of that assessed the current complementary feeding practices in relation to nutrient intakes of Bangladeshi children 6–24 months in Tower Hamlets, providing valuable insight into the current dietary quality of young Bangladeshis. Previous studies have been done on the South Asian population in the UK but nothing specific to the Bangladeshi population in Tower Hamlets. One other participatory study working on complementary feeding with British Bangladeshi families in Tower Hamlets has identified a need for further exploration of the CF practices in order to develop tailored advice for the population, so there is a general void in this area [76]. Limitations include a small sample size, potential recall bias, inability to directly obtain anthropometric weight and height measurements for the infants and children, and recruitment of only English-speaking Bangladeshi women. The small sample size further hindered our ability to draw potential associations between various feeding practices and demographic variables. Furthermore, the lack of tools that assess complementary feeding practices designed for minority ethnic groups in high-income countries limits the ability to make relevant comparisons with other studies that consider either low-income countries or European and Western ethnicities. Using the WHO indicators in high-income countries such as the UK allows powerful comparisons among different ethnicities and provides necessary insight into the implications of poor complementary feeding practices for different populations globally, because household surveys that include dietary diversity usually exclude the early childhood age group and do not use the WHO indicators [77]. Implementing the use of tools such as ICFI or WHO indicators within the healthcare sector provides workers with a simple framework to assess, monitor, and evaluate complementary feeding practices and also allows caregivers to self-assess and improve regularly. Education programs are needed and must be tailored to focus on the Bangladeshi traditions, practices, and cultural dishes and emphasise on the large contribution of the Westernised foods for infants and young children in the UK.

## Conclusions

There is a lack of research exploring complementary feeding practices and nutrient intakes of 6–24-month-old South Asian or Bangladeshi ethnicities in Europe and in the UK specifically. As such, we cannot assume that results from our study are generalisable to the population due to the small sample size; thus, a larger scale study is needed. In this study, the complementary feeding practices and dietary intakes were assessed using several tools. This study showed the following: (1) different aspects of complementary feeding practices of some children were suboptimal; (2) protein intakes were higher than recommendations for all age groups; (3) compared to the RNIs, iron, zinc, and vitamin D intakes were lower while calcium, vitamin C, folate, thiamin, and riboflavin intakes were higher for different age groups. Information from our feasibility study encourages future research on a larger scale to determine if certain modifiable dietary and lifestyle risk factors could be identified for this age group through complementary feeding practices and improved through early intervention.

## Supplementary information

**Additional file 1: Table S1.** Maternal Anthropometric Characteristics. A table that includes some maternal anthropometric data including BMI and BMI class, shown as number and %.

## Data Availability

The datasets generated and analysed during the current study are not publicly available due to need for confidentiality specified by the Research Ethics committee, but an anonymized version is available from the corresponding author on reasonable request.

## Abbreviations

24hDR: 24-h dietary recall
ANOVA: Analysis of variance
BMI: Body mass index
CF: Complementary feeding
CFUI: Complementary feeding utility index
DNSIYC: Diet and nutrition survey of infants and young children
DRV: Dietary reference value
ICFI: Infant and child feeding index
MAD: Minimum acceptable diet
MDD: Minimum dietary diversity
MMF: Minimum meal frequency
NHS: National Health Service
PCHR: Personal child health record
SD: Standard deviation
UK: United Kingdom
WHO: World Health Organization

## Footnotes

1 Median household income for Tower Hamlets was £29,937 in 2018.
